# The effect of tube voltage on scan–rescan reproducibility of compositional plaque volume: technical variability is not true biological change

**DOI:** 10.1093/ehjimp/qyae041

**Published:** 2024-05-13

**Authors:** Francesca Calicchio, Eric Hu, Shawn Newlander, Alexander Van Rosendael, Elizabeth Epstein, Austin Robinson, Samantha R Spierling Bagsic, Jadranka Stojanovska, Jorge Gonzalez, George Wesbey

**Affiliations:** Scripps Clinic, Scripps Health, 9898 Genesee Ave, 92037, La Jolla, CA, USA; Department of Research and Development, Scripps Health, La Jolla, CA, USA; Scripps Clinic, Scripps Health, 9898 Genesee Ave, 92037, La Jolla, CA, USA; Department of Cardiology, Leiden University Medical Center, Leiden, The Netherlands; Scripps Clinic, Scripps Health, 9898 Genesee Ave, 92037, La Jolla, CA, USA; Scripps Clinic, Scripps Health, 9898 Genesee Ave, 92037, La Jolla, CA, USA; Department of Research and Development, Scripps Health, La Jolla, CA, USA; Department of Radiology and Cardiac Imaging, New York University Medical Center, New York, NY, USA; Scripps Clinic, Scripps Health, 9898 Genesee Ave, 92037, La Jolla, CA, USA; Scripps Clinic, Scripps Health, 9898 Genesee Ave, 92037, La Jolla, CA, USA

The majority of coronary computed tomography angiography (CCTA)-based studies focuses on the clinical implications of different types of coronary plaques, with a particular emphasis on low-attenuation plaque (LAP) and its prognostic implications. However, as much as we rely on the different components of plaques, one should be aware that many different technical settings, in particular tube voltage and lumen attenuation, can have a meaningful impact on plaque components.^1^ The software platform employed, as well as the use of fixed vs. adaptive Hounsfield unit (HU) thresholding, may significantly affect plaque volume and composition.^2,3^ Takagi *et al.*^1^ showed that lower tube voltages result in a change in luminal plaque attenuation, with an apparent increase in calcified plaque volume and an apparent decrease in non-calcified volume. Based upon such findings, the Society of Cardiovascular Computed Tomography 2020 consensus document on coronary plaque concluded that serial CCTA scans to assess plaque evolution should be considered only for research purposes.^4^

Therefore, we thought to retrospectively ascertain the effect of tube voltage on plaque volume and components. The ultimate goal is to underline the need for a ‘reference standard’ and not a ‘gold standard’ for performing, analysing, and reporting serial CCTA findings. Defining accuracy and reproducibility is crucial to eventually allow for serial monitoring of coronary plaques over time.

A 256-slice GE Revolution single heartbeat CT scanner was used for this retrospective institutional review board–approved study. Patients with prior coronary revascularization were excluded. Inclusion criteria were two serial CCTA scans performed in the same contrast bolus, two heartbeats apart, at 140 kVp, followed by 100 kVp (first group), or at 100 and 100 kVp (second group). All other technical settings were identical. We investigated the differences in plaque percent diameter stenosis, plaque volume and composition, remodelling index, and percent atheroma volume using the FDA-approved software (CLEERLY). Analysis was blinded between scans 1 and 2. We focused on total plaque volume (TPV), calcified plaque (CP), and non-calcified plaque (NCP) and reported plaque volume in mm^3^. Low-attenuation plaque measurements were too low to draw any conclusions.

We first analysed *n* = 20 clinically referred patients at 140 and 100 kVp (140/100 group) and *n* = 21 clinically referred patients at 100 and 100 kVp (100/100 group). For segmental analysis, suboptimal quality image (Likert score < 3) was excluded.

In the 140/100 kVp group [40% women, mean age 71, coronary artery calcium (CAC) score 590, heart rate (HR) 63], the mean total dose length product (DLP) for the two scans combined was 147.6 mGy-cm. At a segmental level (*n* = 176), mean TPV was unchanged (24 vs. 25 mm^3^; *P* = 0.16). However, TPV at the patient level was significantly different in the 140 vs. 100 group (371 vs. 395 mm^3^; *P* = 0.001). In the 100 kVp subgroup, CP was higher (258 vs. 208 mm^3^; *P* ≤ 0.001), while NCP was significantly lower (137 vs. 162 mm^3^; *P* = 0.008), both at the segmental and patient levels.

In the 100/100 kVp group demographics were as follows: 57% women; mean age, 66; CAC, 47; and HR, 65. Mean total DLP combined was 127 mGy-cm. For this group, the TPV was 94 mm^3^. There was a 2.2% difference (*P* = 0.04) in lumen volume between the two scans. All other measurements were unchanged both at the patient and segmental levels. The per-patient total coronary tree Bland–Altman (BA) bias and the 95% limit of agreement (LOA) in mm^3^ were NCP (−0.4; −5.9, 6.8), CP (1.3; −4.8, 7.4), and TPV (1.7; −6.4, 9.9), with ICCs of 0.999, 0.997, and 0.999, respectively (*Figure 1*).

**Figure 1:**
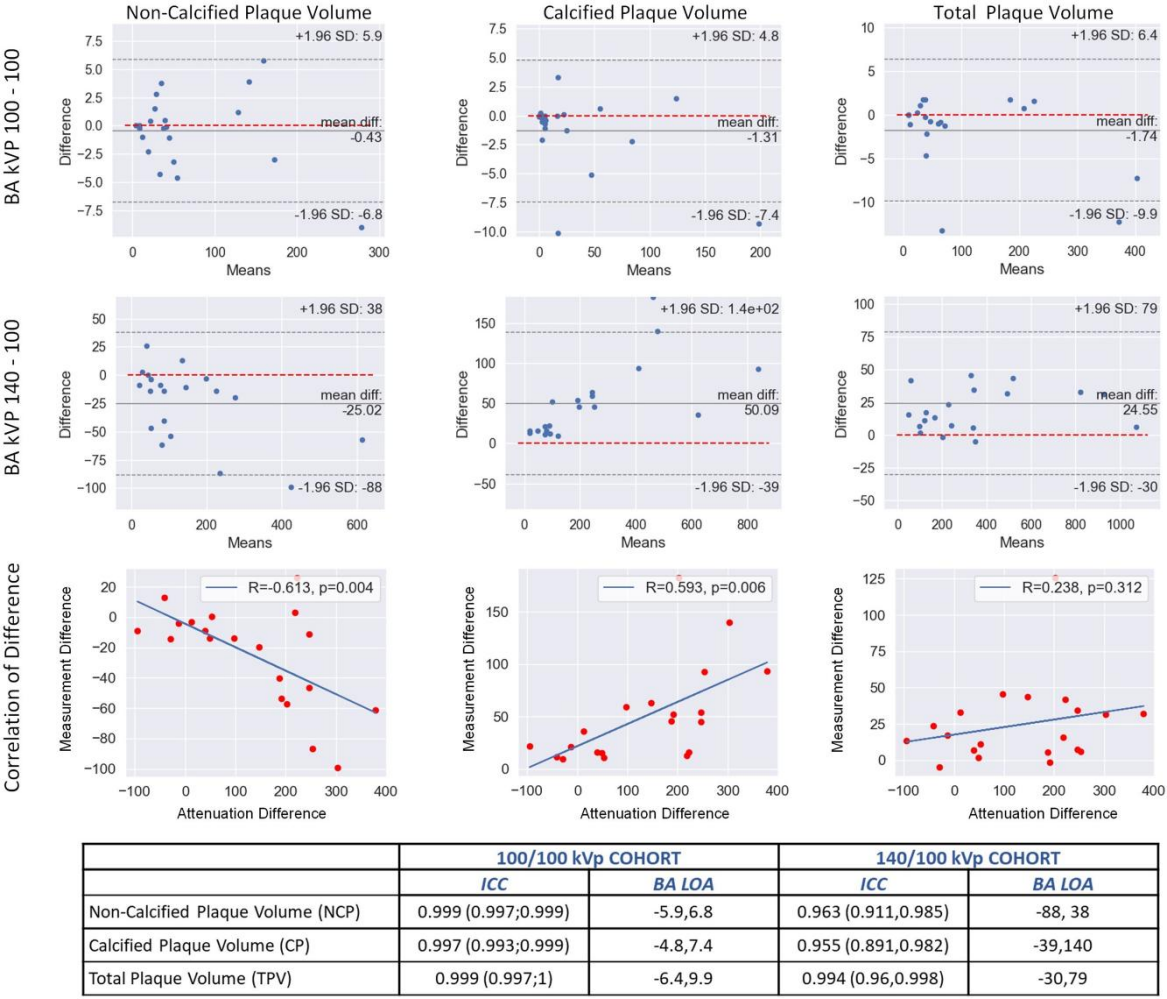
Top panel: Bland–Altman (BA) and correlation plots for non-calcified plaque (NCP), calcified plaque (CP), and total plaque volume (TPV) in the 100/100 vs. 140/100 kVp group (per-patient analyses). Fixed HU threshold was used: LAP < 30 HU, NCP 30–350 HU, and CP > 350 HU. Bottom panel: ICC and BA LOA for NCP, CP, and TPV.

The ascending aortic HU was 520 vs. 653 in the 140 vs. 100 kVp group vs. (*P* = 0.000), with no change in the 100/100 kVp group (677 vs. 691; *P* = 0.223). The increasing ascending aortic HU difference between the 140 and 100 kVp scans correlated with continuous NCP decrease (*r* = −0.61, *P* = 0.004) and CP increase (*r* = 0.59, *P* = 0.006) (*Figures 1* and *2*). The overestimation of CP by 100 kVp resulted in significantly higher TPV because of blooming.^5^

**Figure 2:**
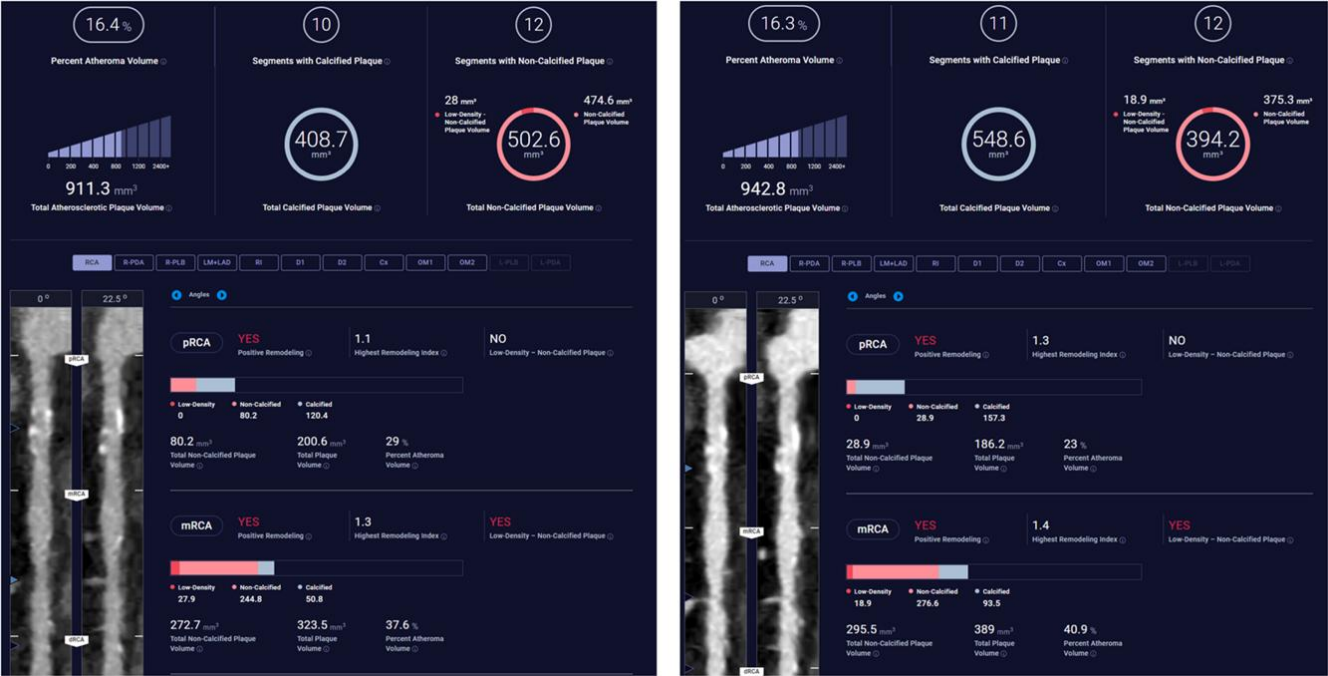
The top row of each acquisition summarizes the total coronary tree compositional plaque measurements. Notice the change in all three compositional plaque volumes for the total coronary tree. In particular, non-calcified plaque volume decreases by 99.4 mm^3^ with a change in kVp from 140 to 100. Total calcified plaque volume increases from 408.7 to 548.6 mm^3^. In combination, this change could be theoretically the same effect one could achieve with statin treatment converting non-calcified plaque to calcified plaque. However, at two heartbeats apart, this represents technical change due to tube voltage. Representative compositional plaque measurements per segment can be found in the lower portion of each report for the proximal RCA and mid RCA.

In the 100/100 kVp group, contrary to the 140 vs. 100 kVp group, we demonstrated that all plaque and stenosis measurements remain unchanged, with >90% lower BA LOA and elimination of bias. The BA 95% LOA from scans 1 to 2 for all plaque components was extremely narrow with very high ICC (*Figure 1*). The results of the 100/100 kVp group demonstrate the high reproducibility of results with quantitative analysis of serial plaque volume. Our results for tube voltage plaque effects are very well aligned with those from Takagi *et al.*^1^

Uniform vendor- and site-specific protocols are needed globally to optimize accuracy and reproducibility for fixed HU threshold analysis in serial CCTA studies. Variability in scan–rescan limits of agreement need to be taken into consideration before assessing biological change.

## Data Availability

The data that support the findings of this study are available on request from the corresponding author of the paper.

## Lead author biography

Dr Calicchio stands as a distinguished cardiologist, holding licences in Italy and the UK. She completed her medical education and fellowship at the University of Pisa, Italy, followed by training at the Royal Brompton Hospital in London, UK. In 2023, she attained her PhD in “Clinical Pathophysiology of Cardiovascular Diseases” at the University of Pisa. Presently, she is a resident and on track to become a cardiology fellow at the Southwest Healthcare in California, USA. She is a research scholar at the Lundquist Institute at Harbor-UCLA and a visiting researcher at the Scripps Clinic in California, USA. Dr Calicchio’s research endeavours range from valvular heart disease to advanced imaging, with a focus on cardiac CT and echocardiography.
